# An embedded method for gene identification problems involving unwanted data heterogeneity

**DOI:** 10.1186/s40246-019-0228-0

**Published:** 2019-10-22

**Authors:** Meng Lu

**Affiliations:** Department of Information Management,Tianjin University, Tianjin, China

**Keywords:** Unwanted heterogeneity, Gene identification, Embedded variable selection

## Abstract

**Background:**

Modern applications such as bioinformatics collecting data in various ways can easily result in heterogeneous data. Traditional variable selection methods assume samples are independent and identically distributed, which however is not suitable for these applications. Some existing statistical models capable of taking care of unwanted variation were developed for gene identification involving heterogeneous data, but they lack model predictability and suffer from variable redundancy.

**Results:**

By accounting for the unwanted heterogeneity effectively, our method have shown its superiority over several state-of-the art methods, which is validated by the experimental results in both unsupervised and supervised gene identification problems. Moreover, we also applied our method to a pan-cancer study where our method can identify the most discriminative genes best distinguishing different cancer types.

**Conclusions:**

This article provides an alternative gene identification method that can accounting for unwanted data heterogeneity. It is a promising method to provide new insights into the complex cancer biology and clues for understanding tumorigenesis and tumor progression.

## Background

Many variable selection methods have been proven to be successful in handling high-dimensional data in the past few decades. However, most of these methods assume the data are independent and identically distributed (i.i.d.), whose applications are severely limited. In most real-world applications, we are quite often facing the heterogeneous data violating the assumptions [1]. For instance, the gene expression data are usually found confounded by “batch effects” in bioinformatics [2–4], which refers to the systematic error generated while the samples are probed by multiple batches of platforms [5–7]. In many high-throughput biological experiments, technical noise will significantly influence the subsequent data analyses [8]. Using technical replicate measurements is a common way to reduce the impact of technical noise on gene expression analysis. However, technical replicates or batch effects will definitely cause the data samples have group structures, resulting in heterogeneous data for which the traditional variable selection methods designed for generic data are not suitable. These experimental or technical factors are usually known and can be easily adjusted. Nevertheless, gene expression data typically suffer from other additional unknown factors that can induce more complex data heterogeneity. It is not the truth that all of these heterogeneous factors are unwanted in association analysis or variable selection problems. Sometimes a certain known/observed heterogeneous factor could be of research interest and the others are unwanted. For example, when one would like to know the variables that can best describe the data grouping structure. In this case, only those heterogeneous factors except the grouping factor are unwanted. While in some other circumstances, the heterogeneous factors are unwanted if none of them is of interest. For example, some gene expression data are confounded by batched effects and some other unknown factors, and the corresponding gene identification problem is to seek those genes discriminative in the related disease phenotype. In this case, the factor of research interest is the disease phenotype and all the other heterogeneous factors confounding the gene expression data are unwanted. Therefore, the specific problems and the factors of interest determine whether the gene expression heterogeneity factors are wanted or unwanted in the association analysis. In either case, if the unwanted heterogeneity is not taken into account, it would lead to spurious results by directly applying conventional variable selection methods to gene identification problems involving heterogeneous data. Moreover, it is even more challenging to adjust the unwanted heterogeneity if unknown heterogeneity are involved.

Existing methods proposed for feature selection on heterogeneous data deal with those data generated from multiple views or sources that describe the same set of samples [9–12]. The corresponding data heterogeneity generated from multiple views or sources are taken advantage to improve the results learned from any single data source. This is usually completed by formulating variable selection problem as an multiple-task learning or integrative learning problem. The variable selection strategies in most of these methods are inspired by sparse learning based models [13, 14] or sparse principal component analysis [15, 16]. However, this article studies different problems in which the performance of variable selection is hurt by the data heterogeneity that should be removed. Most previous related work addressing the heterogeneity issue focuses on adjusting the observed data heterogeneity. However, the data analysis results in bioinformatics have proven to be significantly influenced by the complex data heterogeneity arising from unknown heterogeneity factors [17, 18]. Rare attentions have been paid to the adjustment of this kind of data heterogeneity. Surrogate variable analysis (SVA) [17] have been proposed by Leek and Store to adjust the unknown data heterogeneity for statistical analysis. This method lacks model predictability and has the variable redundancy issue as a filtering variable selection method [1]. Therefore, this article proposes an effective embedded variable selection method that allows for model prediction from a sparse learning perspective [19]. Our method is capable of adjusting the unwanted heterogeneity no matter they are known or unknown. Moreover, it is also suitable for both unsupervised and supervised variable selection problems. We will study three different unsupervised and supervised gene identification problems to investigate its performance based on either gene expression benchmark data or real-world RNA-Seq gene expression data.

## Methods

### Data modelling

We denote the gene expression data by *X*, which contains *n* samples and *p* variables. The group information causing the heterogeneity is represented by a binary indicator matrix $G\in \mathbb {R}^{n\times g}$ whose element *G*_*ij*_ with value ‘1’ indicates that the *i*-th sample belongs to the *j*-th group and vice versa. In different kinds of variable selection problems, the group factor can play different roles contingent on the corresponding interested factors. For instance, we have the unsupervised variable selection problems aiming to select the variables that can best determine the data group structure. In this problem, our factor of interest is *G* and we model *X* by:

$$X = G\mathbf{\eta}+ \mathbf{\epsilon}, $$

where **ε**∈*N*(0,*σ*^2^*I*) represents the i.i.d. noise and $\mathbf {\eta }\in \mathbb {R}^{g\times g}$ reflects the group factor effect on each variable. While in the supervised variable selection problems, additional observed variables denoted by *Y* are introduced and affected by the variables of *X*. The factors of interest is *Y* here and the association between *Y* and the variables of *X* is what we want to study. In this case, we model *X* by :

$$\begin{array}{@{}rcl@{}} X = Y\mathbf{\gamma}+G\mathbf{\eta}+\mathbf{\epsilon}, \end{array} $$

in which **γ** reflects the effect of *Y* on each variable.

In both types of the above problems, there may also involves some other unaware factors causing unknown heterogeneity sometimes. The unknown heterogeneity refers to the variation patterns caused by the unknown factors that are not explicitly included in the data model. Let *F*={*F*_*k*_:1≤*k*≤*K*} denote the *K* unknown factors. Certainly, they should be regarded as uninterested factors and included in the data models. Thus, we correct the model for unsupervised problems as:

1 $$\begin{array}{@{}rcl@{}}  X = G\mathbf{\eta}+ F\mathbf{\phi}+ \mathbf{\epsilon}, \end{array} $$

and the model for supervised problems as:

2 $$\begin{array}{@{}rcl@{}}  X = Y\mathbf{\gamma}+G\mathbf{\eta}+ F\mathbf{\phi} +\mathbf{\epsilon}. \end{array} $$

Apparently, the analysis of true relations between the factors of interest and each variable should take the unwanted heterogeneity into account. In unsupervised problems, the unwanted heterogeneity is caused by those uninterested factors referring to *F*. While in supervised problems, those uninterested factors refer to {*G*,*F*}.

### Variable selection

Our variable selection strategy contains two main stages: (1) adjust the unwanted heterogeneity and create a new data set *X*_*a*_. The effects of the factors of interest are thus represented by the variables of the new data set; (2) based on the adjusted data set *X*_*a*_, select the variables associated with the factors of interest.

It is impossible to directly estimate the unknown factors *F* for the first stage. We take the procedure similar to SVA [17] to estimate the unknown factors. After removing the effects from the uninterested factors, we obtain a new data set whose variation is only determined by the factors of interest. We show the detailed steps of the first stage as below.

**Stage 1: Creating a new adjusted data set** ***X***_***a***_ **.**

*1. Remove the effects from the known factors.*

We first reformulate both model (1) and (2) in a general form:

3 $$\begin{array}{@{}rcl@{}}  X = N\mathbf{\omega}+F\mathbf{\phi}+\mathbf{\epsilon}, \end{array} $$

where *N* dentotes the known factors which refer to *G* for model (1) and {*Y*,*G*} for model (2). Let ℜ_*N*_ denote the column space of *N*. Correspondingly, let *R*_*N*_ denote the residual operator of *N* that projects onto the orthogonal complement of ℜ_*N*_, which is denoted by *I*−*N*(*N*^*T*^*N*)^−1^*N*^*T*^. By multiplying *R*_*N*_ to both sides of model (3), we have:

$$\begin{array}{@{}rcl@{}} R_{N}X&=& R_{N}N\mathbf{\omega} + R_{N}F\mathbf{\phi} + R_{N}\mathbf{\epsilon} \\ &=& R_{N}F\mathbf{\phi} + R_{N}\mathbf{\epsilon}. \end{array} $$

Through projecting *X* onto the orthogonal complement of ℜ_*N*_, we can remove the effects from the known factors *N*.

*2. Extract surrogate factors h*_*k*_.

It is challenging to directly estimate *F*. In this step, we estimate surrogate factors *h*_*k*_(1≤*k*≤*K*) instead to represent the residual heterogeneity *R*_*N*_*F***ϕ**. We can apply any factor analysis method on *R*_*N*_*X* to produce *h*_*k*_. We consider Singular Value Decomposition (SVD) here to remove the arbitrary.

*3. Estimate unknown factors F*_*k*_.

We estimate each surrogate factor *h*_*k*_ based on the following steps: 1) select several variables of *X* most associated with *h*_*k*_;2) conduct SVD on the set constructed by the selected variables and return the eigenvectors *e*_*j*_(1≤*j*≤*n*);3) find *j*^∗^=*argmax*_1≤*j*≤*n*_*cor*(*e*_*j*_,*h*_*k*_) and set $\phantom {\dot {i}\!}F_{k}=e_{j^{*}}$.

*4. Determine the uninterested factors U.*

We set *U* to *F* for the unsupervised cases and to {*G*,*F*} for the supervised cases.

*5. Build a new adjusted data set X*_*a*_.

Both model (1) and (2) are represented by a general form:

4 $$\begin{array}{@{}rcl@{}}  X = M\mathbf{\beta}+U\mathbf{\alpha}+\mathbf{\epsilon}, \end{array} $$

where *M* represents the factors of interest. It refers to *G* for model (1) and *Y* for model (2) respectively. Then, we assume that the true relations between the variables and the factors of interest hide in an adjusted data set *X*_*a*_. By removing the unwanted heterogeneity, we calculate *X*_*a*_ as *X*−*U***α**.

**Stage 2: Scoring variables**

We employed an optimal scoring idea [20] to estimate the true effects of variables to score the data variables associated with the factors of interest *M*. We formulate the following variable selection problem with model robustness:

5 $$\begin{array}{@{}rcl@{}}  \min_{B,\Theta} \frac{1}{n}||X_{a}B-M\Theta||_{F}^{2} + \lambda \varphi(\mathbf{b})\\ s.t. \quad \Theta^{T}M^{T}M\Theta=I, \end{array} $$

where *φ* sums over a vector of row norms (i.e, **b**=[||**b**_1_||_2_,…,||**b**_*p*_||_2_]^*T*^ where **b**_*j*_(1≤*j*≤*p*) denotes the *j*-th row of the *p*×*l* projection matrix *B*). The *l*_2_ norm of the *j*-th row vector of *B* reflects the influence of the *j*-th variable of *X* on *M*. The variable selection is allowed based on the row norms of *B* with an appropriate choice of *λ*. We update all the unknown variables iteratively until convergence to find the solutions of problem (5).

In the *t*+1-th iteration, we update *Θ*^*t*+1^ given fixed *B*^*t*^ by solving the subproblem:

$$\begin{array}{@{}rcl@{}} \min_{\Theta} \frac{1}{n}||X_{a}B^{t}-M\Theta||_{F}^{2} \\ s.t. \quad \Theta^{T}M^{T}M\Theta=I. \end{array} $$

We introduce a new variable *Θ*^′^ to denote $\left (M^{T}M\right)^{\frac {1}{2}}\Theta $ and then solve the corresponding optimization problem with respect to *Θ*^′^. By doing SVD of $\left (M^{T}M\right)^{-\frac {1}{2}}M^{T}X_{a}B^{t}$, we obtain the optimal *Θ*^′^.

Given *Θ*^*t*^, we can update *B* by solving the subproblem:

$$\begin{array}{@{}rcl@{}} \min_{B} \frac{1}{n}||X_{a}B- M\Theta^{t}||_{F}^{2} +\lambda \varphi(b). \end{array} $$

Since the *φ* function is a non-decreasing and concave function of the *l*_2_ norm of row vectors of *B*, this is a regularized regression problem which can be solved by iterative reweighted *l*_2_ algorithm [21]. Despite the convergence rate may be slow, the updating rules of this algorithm are closed-form and simple. In detail, the iterative updates are:

$$\begin{array}{@{}rcl@{}}  &B^{(t+1)}= W^{t}X^{T}\left(\lambda nI+XW^{t}X^{T}\right)^{-1}M\Theta^{t},\\ &w_{j}^{(t+1)}= \frac{\partial\varphi(B)}{\partial||\mathbf{b}_{j}||_{2}^{2}}\big|_{B=B^{(t+1)}}= \frac{1}{2||\mathbf{b}^{(t+1)}_{j}||_{2}}, \end{array} $$

in which $W=\text {diag}\left (w_{1}^{-1},\ldots,w_{p}^{-1}\right)$. Since the influences of the variables on the factors of interest are reflected by the *l*_2_ norm of the corresponding row vectors of *B*, we select variables by ranking all the variables in a decreasing order based on them.

In summary, the whole detailed procedure for variable selection of our method is given by Algorithm 1. If it needs *r* iterations to converge, the time complexity of this algorithm is *O*(*r*(*n*^2^*p*+*n*^3^)). Usually, it takes a few iterations to converge based on our experience. It is worth to note that the algorithm is desirable for high-dimensional data because it scales linearly in the number of variables. We name our method as sparse optimal scoring with adjustment (SOSA) considering we use a sparse optimal scoring strategy to select variables based on the adjusted data. 
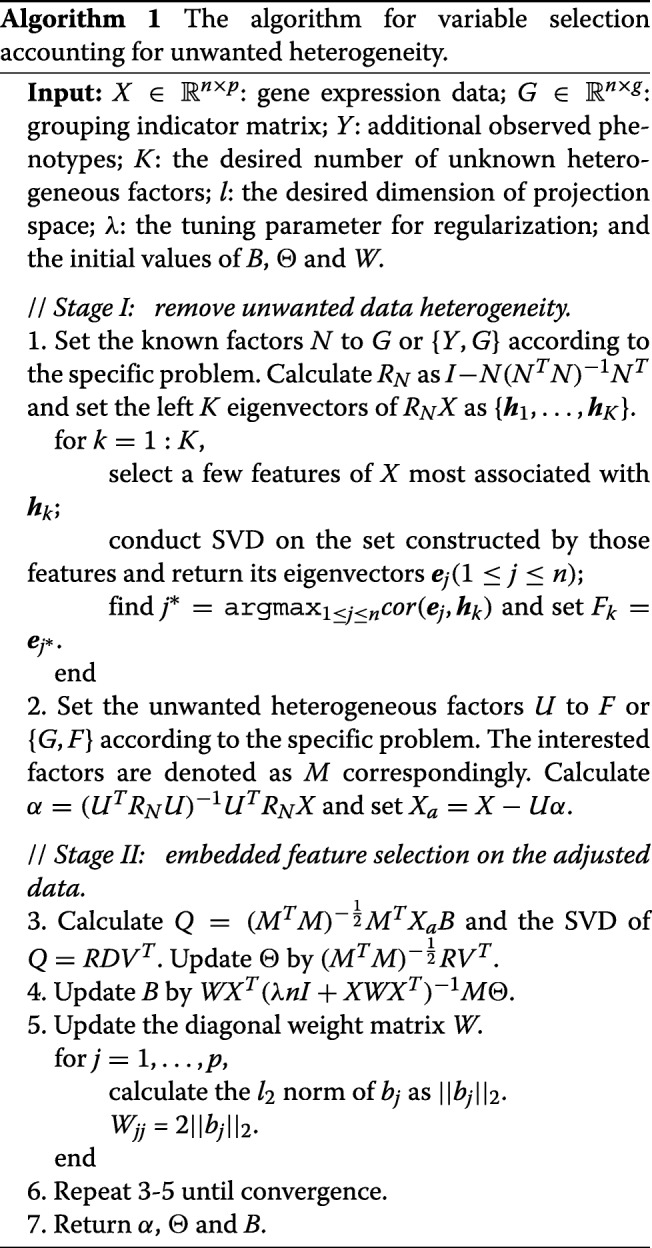


## Results

We studied three different unsupervised or supervised gene expression analysis problems to investigate the performance of our method. The first problem is unsupervised and studied on spike-in gene expression data, while the last two problems are supervised and studied on real-world gene expression data. The performance in gene identification while taking care of unwanted heterogeneity is evaluated by comparing with state-of-the-art methods for each problem.

### Evaluation criteria

We evaluate and compare the gene identification performance of all the methods based on the specificity and sensitivity of the method’s ability to detect the differentially expressed genes. Particularly, we consider the number of true positives and false positives as well as the receiver operating characteristic (ROC) curve. These criteria are only suitable for the experiments in which the true differentially expressed genes are known.

Given a top-N ranking list of the identified genes obtained from a method, *♯true* *positives* is the number of true genes that appear in the top-N ranking list and *♯false* *positives* is the number of false genes that appear in the top-N ranking list. Correspondingly, the true positive rate and false positive rate are:

$${} \begin{aligned} &\text{True positive rate} = \frac{ \sharp true\; positives }{ \sharp true\; genes},\\  &\text{False positive rate} = \frac{\sharp false\; positives }{ \sharp false\; genes}, \end{aligned}  $$

where *♯true* *genes* is the number of true genes which are known truly associated with the factor of interest and *♯false* *genes* is the number of false genes which are known falsely associated with the factor of interest. The receiver operating characteristic (ROC) curve is plotted based on the calculated true positive rate (sensitivity) and false positive rate (1-specificity). A method with the largest area under curve (AUC) achieves both the highest sensitivity and specificity.

### Unsupervised differential gene expression analysis in a spike-in study

This study attempts to find the genes that are differentially expressed in different tissues based on the benchmark data set from the spike-in experiment [22]. It belongs to unsupervised differential gene expression analysis. In this spike-in experiment, a series of 14 human cRNA fragments were spiked-in at known concentration ratings from 0 to 1024 pM, which are the spiked probe sets/genes. For each of the 14 array groups, the cRNAs were spiked-in at different concentrations. In each array group, there were three replicates except that there are two array groups having 12 replicates. The detailed arrangement of concentrations of the 14 cRNAs for each group can be found in [23]. In total, there are 59 arrays belonging to 14 array groups in the spike-in data. Each array contains 12,626 probe sets and 14 of them are spiked-in. Since Cope et al. found two more genes with similar patterns to the spiked genes [22], our goal here is to detect those 16 genes differentiating the 14 array groups. In other words, we want to discover those genes most representative of the biological variation from different tissues and meanwhile most resilient to the technical noise hidden among the technical replicates.

**Fig. 1:**
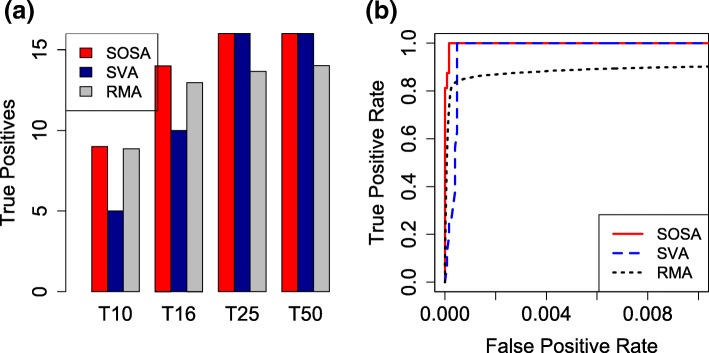
**a** Comparison of the number of true positives (i.e, hits) in top 10, 16, 25 and 50 rankings for SVA, RMA and our SOSA in spike-in study; **b** Comparison of ROC curves for SVA, RMA and our SOSA in spike-in study

**Fig. 2:**
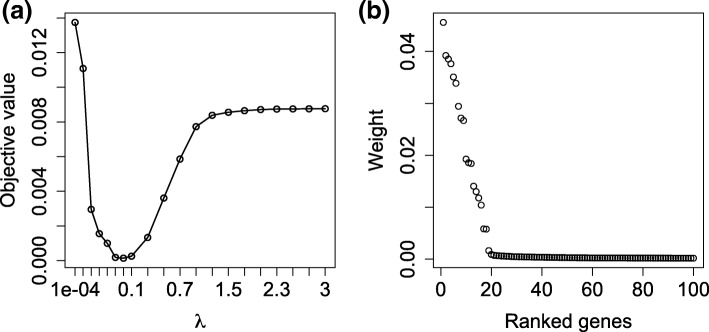
**a** A plot of average objective values for the testing data at 20 different *λ*s in the range between 0.0001 and 3; **b** A plot of corresponding row-norms of *B* for the top 100 ranked genes in spike-in data

We compared three methods accounting for the heterogeneity or not. Robust Multi-array Average (RMA) is the most popular method for gene expression analysis but taking no heterogeneity into account. It summarizes the replicate measurements to a gene expression value [24], which however loses the technical variation information hidden in the replicates. We ranked the genes based on their average fold-change in RMA-summarized gene expression values. Another two representative methods taking care of the heterogeneity we considered here are SVA and SOSA. SVA uses the estimated uninterested factors as covariates and conducts association analysis for each individual gene. The genes are ranked in the increasing order of their *p*-values. In contrast, our SOSA ranked the genes based on the *l*_2_ norm of their corresponding row vectors of the estimated *B*. We then calculated the respective true positives and false positives based on the rankings of the three methods. The number of their true positives appearing in the top 10, top 16, top 25 and top 50 ranking lists are shown by Fig. 1a. The reason we investigate the top 16 ranking list is that the number of the ground-truth genes is 16. We found that SOSA always identified the most true positives in all the cases. Fig. 1b further showed a more comprehensive investigation by ROC curves, in which our SOSA achieved the highest sensitivity and specificity while RMA the lowest. The superiority of our method is further validated. We select variables via choosing an appropriate *λ* to force the corresponding row-norms of *B* for most genes close to 0. The choice of *λ* was done by cross validation in which we sequentially select one array out of each array group to build the testing data and the rest to build training data. The average objective values calculated for the testing data using the corresponding estimated parameters at 20 different values of *λ* are shown by Fig. 2a. We chose the best *λ* as 0.001 that corresponds to the smallest objective value. Usually, the range of the best *λ* is expected falling in [0.0005,0.01]. Figure 2b showed the corresponding top-100 ranking of genes based on their corresponding row-norms of *B*. We found that the top-ranked 16 genes matched the ground-truth exactly.

### Supervised differential gene expression analysis in a gender study

In this experiment, we study a supervised gene identification problem which is first conducted by Vawter et al. [25]. It tries to find those genes differentially expressed in human brain with respect to the gender. In this problem, the samples are clearly clustered by laboratory and chip type because they were sent to different laboratories measured by one of these two types of chips. The interested effects related to the gender were blurred by these strong batch effects and consequently the analysis results will be significantly influenced. For this problem, our goal is to find those genes that can best differentiate the samples from different genders. Moreover, these genes should also be resilient to the unknown effects and the known batch effects. The samples were taken post-mortem from the brains of 10 individuals including 5 men and 5 women in this study. Specifically, for each individual, the experiment takes three samples from different regions of its brain. Each sample was sent to three laboratories for analysis based on either Affymetrix HG-U95Av2 or Affymetrix HGU95A platform. Since there are 6 missing chips in the 90 combinations, we have 84 collected chips in total. The processed data set keeps the 12,600 probe sets shared by the two platforms. RMA is used to measure the gene expression values for the 84 chips.

**Fig. 3:**
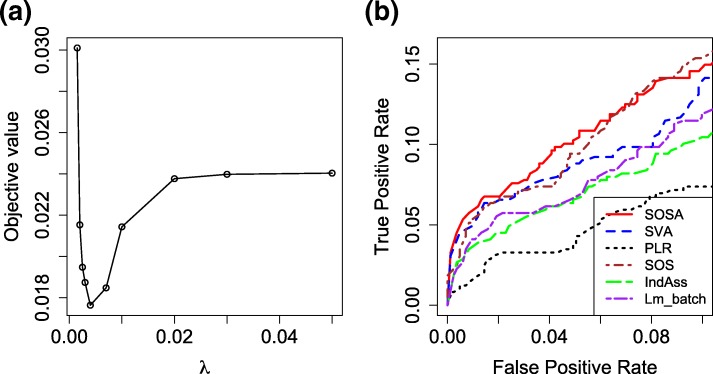
**a** A plot of average objective values for the testing data at 10 different *λ*s in the range between 0.0001 and 0.05; **b** Comparison of ROC curves for PLR, SOS, IndAss, Lm _batch, SVA and our SOSA in the gender study

We compared six representative methods: individual gene association analysis (IndAss), penalized logistic regression (PLR), the standard sparse optimal scoring (SOS), linear model adjusting batch effects (Lm _batch), SVA and our SOSA, to identify those genes that can best differentiate the samples from different genders. Their sensitivity and specificity in gene selection were investigated. PLR, IndAss and SOS run directly on the gene expression data without accounting for the unwanted heterogeneity. The ‘glmnet’ package was employed to implement PLR with the parameter chosen by cross validation. We ranked the genes based on the absolute values of the regression coefficients in a decreasing order. Linear model was also employed here to adjust the known batch effects, which however is not able to adjust the unknown heterogeneous factors. We also considered two alternative methods accounting for the heterogeneity: SVA and our SOSA. We considered three unknown factors here. By taking the lab/chip factor and the estimated unknown factors as covariates, SVA did the association analysis for each individual gene and then ranked the genes based on their *p*-values in an increasing order. In contrast, our SOSA ranked the genes based on the *l*_2_ norm of their corresponding row vectors of the estimated *B*. The parameter *λ* in SOSA was chosen by leave-one-out cross validation. Figure 3a showed the plot of average objective values for the testing data at 10 different values of *λ* falling in the range between 0.0001 and 0.05. The best *λ* was chosen as 0.004 which corresponds to the minimum average objective value across all the folds. We treat all the 488 genes from the *X* and *Y* chromosomes as candidate positive controls considering most genes differentially expressed with respect to the gender will be located on the *X* and *Y* chromosomes. For the negative controls, we use the 799 housekeeping genes claimed by [26]. The ROC curves for all the six methods are shown by Fig. 3b, in which the superiority of our SOSA is validated since it almost always reached the highest sensitivity and specificity. Table 1 showed the number of true positives and false positives in the top 20, 60, 80, 180 for each method. We observed that those methods with adjustment can identify more *X*/*Y* genes or less housekeeping genes than the unadjusted ones, which demonstrates their significant improvement on the gene selection performance. Benefiting from the model stability accomplished by the regularization of the gene coefficients, our SOSA achieved better performance than SVA approach. We also observed that SOSA is superior to Lm _batch since it can adjust both the known batch effects and the unknown heterogeneous factors. For those methods without adjustment, SOS and IndAss achieved higher sensitivity than PLR by correctly detecting more *X*/*Y* genes, but IndAss has lower specificity since it wrongly identified much more housekeeping genes than the other two methods.

**Table 1:** Comparison of the number of true positives and false positives found for PLR, SOS, IndAss, Lm _batch, SVA and SOSA in the gender study

		Top 20		Top 60		Top 80		Top 180	
		TP	FP	TP	FP	TP	FP	TP	FP
Unadjusted	PLR	3	1	4	2	4	2	7	7
	SOS	9	0	15	4	17	4	25	8
	IndAss	9	2	14	5	17	7	24	20
Adjusted	Lm _batch	9	2	13	5	17	6	23	13
	SVA	16	1	22	7	25	8	30	15
	SOSA	16	1	24	4	26	5	32	13

### Supervised differential gene expression analysis in pan-cancer study

The two studies above investigated the gene identification performance of our method on heterogeneous gene expression benchmark data with ground-truth genes known. In this experiment, we explore the gene identification performance of our method as well as the interpretation of those identified genes in the application to a pan-cancer study. This experiment aims to identify a set of genes differentially expressed across five common types of cancer that are clinically attractive as diagnostic biomarkers or therapeutic targets based on The Cancer Genome Atlas (TCGA) gene expression data. Particularly, we use the publicly available RNA-Seq (HiSeq) PANCAN data set from the UCI machine learning repository (https://archive.ics.uci.edu/ml/datasets/gene+expression+cancer+RNA-Seq##), which is a random extraction of RNA-Seq gene expressions of patients having five types of cancer: Breast (BRCA), Colon (COAD), Kidney (KIRC), Lung (LUAD) and Prostate (PRAD) from the TCGA data portal. This extracted and preprocessed data set contains 801 patients with 20,531 genes measured in total. Among them, 300, 78, 146, 141 and 136 patients have BRCA, COAD, KIRC, LUAD and PRAD respectively, which implies that the samples are relatively balanced across different types of cancer.

To identify the set of genes that can best classify these five types of tumor, we considered five methods including three typical methods without accounting for any unwanted heterogeneity: PLR, SOS, IndAss and two methods that are able to adjust for the unwanted heterogeneity: SVA and SOSA. We separated the data samples into training samples and testing samples by a 5-fold cross validation. The differentially expressed genes were identified using these methods on the training data respectively. Their utility was investigated by evaluating the corresponding classification performance on the test samples using them based on 1-*NN* classifier. The average classification testing error rates for each method using their identified top 10, 30, 50, 100, 200, 300, 400 genes are shown by Table 2. We also show the classification testing error rate using all the genes on the test data as the baseline. The different types of cancer can be well classified with a 99.5% classification accuracy using all genes. The utility of the respective top 30 genes identified by PLR, SOS and SOSA are competitive to the baseline. As filtering methods, IndAss and SVA identified pretty worse sets of top *N* genes than the others for *N* less than 50 due to lack of considering the combining effects of the identified genes. With *N* larger than 200 but not too large, the top *N* genes identified by each of the methods can lead to competitive or higher classification performance than the baseline. Among these methods, our SOSA can even achieve 99.9% classification accuracy with its top 100 identified genes. Overall, our SOSA can identify the most discriminative genes comparing to other methods at each fixed number of selected genes. And the superiority is even higher as the number of selected genes goes small.

**Table 2:** Comparison of the average classification test error (%) in 5-fold cross-validation using different number of top genes selected by: (1) a set of methods without adjustment for heterogeneity: PLR, SOS, IndAss; (2) another set of methods accounting for heterogeneity: SVA and SOSA respectively in the Pan-Cancer study

		T10	T30	T50	T100	T200	T300	T400
Unadjusted	PLR	1.9	0.4	0.4	0.4	0.1	0.1	0.1
	SOS	3.1	0.4	0.3	0.3	0.3	0.3	0.3
	IndAss	4.5	1.5	1.8	0.1	0.3	0.1	0.1
Adjusted	SVA	4.3	1.5	1.3	0.6	0.3	0.3	0.1
	SOSA	1.1	0.5	0.3	0.1	0.1	0.1	0.1
Baseline	Using all 20,531 genes	0.5						

In addition to examination of the classification performance of the identified genes, we care much more about the interpretation of the role of these genes in distinguishing the five cancer types as well as their involved biological processes. Specifically, we found the union of the top 200 genes identified from each fold of the partitions. The final top 20 genes in the union set were: MAB21L1, HAND1, SFTA2, SFTPA2, CDV3, NBL1, SFTA3, KLK1, HNF1A, NKX1-2, NOV, KLK2, SFTPC, PQLC3, TCF20, NAPRT1, POU3F2, CDH15, SCGB3A1 and GP9.

MAB21L1 belongs to the conserved male abnormal gene family 21, which is described as a transcription factor in cell fate determination [27]. Heart and neural crest derivatives expressed 1 (HAND1) is a basic helix-loop-helix transcription factor, and plays a very important role in the development and differentiation of heart and nervous system. As a developmental regulator, HAND1 is silenced in over 90% of human primary colorectal tumors [28]. Four surfactant genes (SFTA2/3, SFTPA2, and SFTPC) were identified in the top 20. They were highly expressed in lung cancer and low in all other tumors. CDV3 was documented as an unidentified gene in breast cancer [29], whose expression correlated to the expression of Her2 and the sensitivity of photon-irradiation and simultaneous PTX-treatment in breast cancer [30]. Three kallikrein genes (KLK1/2/3) were identified in the top 30. The risk of prostate cancer has been reported associated with single-nucleotide polymorphisms (SNPs) located in the genes coding for PSA (KLK3) [31] and hK2 (KLK2) [32]. The classical cadherins (CDHs) are a superfamily of transmembrane glycoproteins involved in calcium-dependent cell–cell adhesion in embryonic development and epithelial tissues. CDHs are also associated with signaling, mechanotransduction, cancer progression, and tissue morphogenesis, many of which are related to cancer [33].

**Fig. 4:**
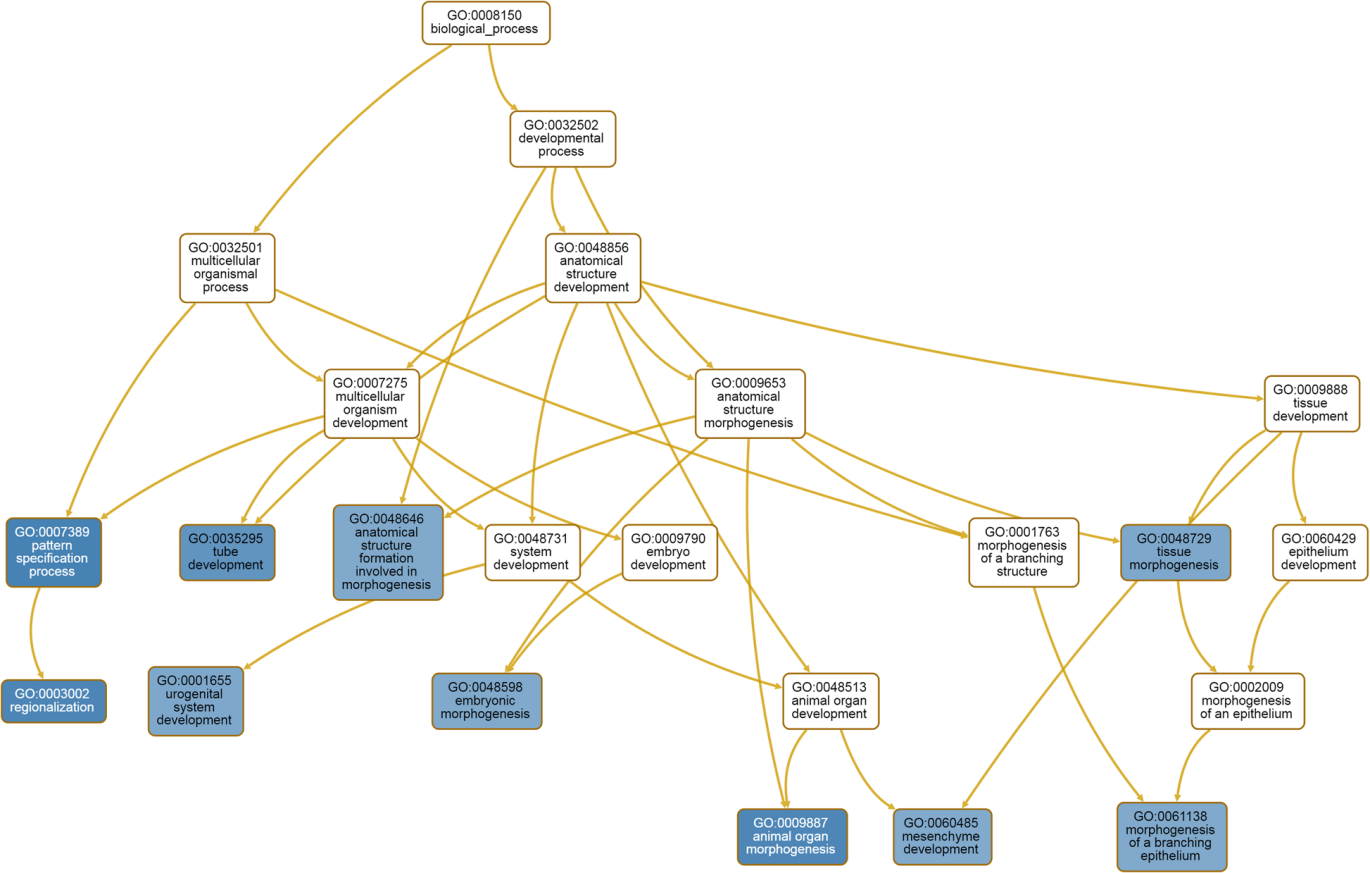
An acyclic graph showing significantly enriched GO categories under biological process. The top 10 GO terms are denoted as the blue nodes in the graph

We also performed gene ontology (GO) analysis of the top 200 genes using WebGestalt tool [34]. The top 10 highly enriched biological processes with GO terms are listed in Table 3 and the corresponding topology is shown by Fig. 4. Each gene ontology category is a node in the graph. GO categories in blue nodes are the top 10 enriched GO categories while the others are their non-enriched parent categories. It is clear that many of these genes are involved in the biological processes of development or morphogenesis of multicellular organism, organ, embryo and tissue, which may provide some clues for understanding tumorigenesis and tumor progression as well as new insights into the complex cancer biology.

**Table 3:** Top 10 Enriched gene ontology (GO) terms for the most discriminative 200 genes from the pan-cancer classification of all 801 samples having BRCA, COAD, KIRC, LUAD and PRAD

Gene Ontology (GO) terms	*P*-value
Pattern specification process	2.6e-11
Regionalization	9.6e-11
Animal organ morphogenesis	1.6e-10
Tube development	1.1e-9
Urogenital system development	1.6e-8
Tissue morphogenesis	1.8e-8
Morphogenesis of a branching epithelium	2.2e-8
Anatomical structure formation involved in morphogenesis	2.5e-8
Embryonic morphogenesis	2.8e-8
Mesenchyme development	3.5e-8

## Discussion

The results from our experiments on three unsupervised and supervised gene identification problems have shown the superiority of our method in handling unwanted data heterogeneity issue. The first problem studied a representative application of finding the differentially expressed genes from gene expression data with batch effects in unsupervised circumstance. Our method performs better than RMA and SVA since it is capable of taking care of the unwanted heterogeneity as well as variable redundancy. The second problem studied a typical application of identifying differentially expressed genes from gene expression data with batch effects in supervised circumstance. Our method performs better in the sensitivity and specificity in variable selection than other state-of-the-art methods including penalized logistic regression model, simple statistical model for individual gene association analysis, sophisticated statistical model capable of accounting for data heterogeneity and linear model accounting for batch effects. This benefit from our two-stage embedded variable selection strategy. The third problem studied a general case of supervised gene expression analysis where the batch effects are unknown. Our method can achieve the highest classification accuracy comparing to other methods using the same number of discriminative genes. In summary, our method has wide applications in various kinds of gene identification problems.

## Conclusions

We proposed sparse optimal scoring with adjustment (SOSA) for gene identification on heterogeneous data involving unwanted heterogeneity. Particularly, our method is able to account for the unknown and unwanted heterogeneity that blurs the true signals of genes. The results from the first two studies on the batched gene expression data demonstrate that those methods accounting for the unwanted heterogeneity can significantly improve the gene identification performance. Moreover, our method is also superior to the statistical models capable of adjusting data heterogeneity because of the model stability and the ability of prediction on new samples. The results from pan-cancer study further validated the superiority of our method in identifying the genes discriminative in different cancer types to other state-of-the-art methods. The biological interpretation of the results provides new insights into complex cancer biology and clues for understanding tumorigenesis. In view of the commonly existing data heterogeneity issue such as batch effects in bioinformatics, our SOSA can also be adapted and serves as a promising tool to remove the unwanted heterogeneity prior to downstream analysis for many other biomedical applications such as cell population diversity in single-cell landscapes and so on.

## Abbreviations

AUC: Area under curve
FDR: False discovery rate
IndAss: Individual gene association analysis
Lm _batch: linear model adjusting batch effects
PLR: Penalized logistic regression
RMA: Robust multi-array average
ROC: Receiver operating characteristic
SOS: The standard sparse optimal scoring
SOSA: Sparse optimal scoring with adjustment
SVA: Surrogate variable analysis
SVD: Singular value decomposition

## References

[1] Li J, Cheng K, Wang S, Morstatter F, Trevino RP, Tang J, Liu H. Feature selection: A data perspective. arXiv preprint **arXiv:1601.07996**. 2016.

[2] Chen C, Grennan K, Badner J, Zhang D, Gershon E, Jin L, Liu C (2011). Removing batch effects in analysis of expression microarray data: An evaluation of six batch adjustment methods. PLoS ONE.

[3] Almeida A, Paul JT, Magdelenat H, Radvanyi F (2004). Gene expression analysis by real-time reverse transcription polymerase chain reaction: influence of tissue handling. Anal Biochem.

[4] Ma Y, Dai H, Kong X (2012). Impact of warm ischemia on gene expression analysis in surgically removed biosamples. Anal Biochem.

[5] Bakay M, Chen YW, Borup R, Zhao P, Nagaraju K, Hoffman EP (2002). Sources of variability and effect of experimental approach on expression profiling data interpretation. BMC Bioinformatics.

[6] Boedigheimer MJ (2008). Sources of variation in baseline gene expression levels from toxicogenomics study control animals across multiple laboratories. BMC Genomics.

[7] Fare TL (2003). Effects of atmospheric ozone on microarray data quality. Anal Chem.

[8] Glaab E, Schneider R (2015). Repexplore: addressing technical replicate variance in proteomics and metabolomics data analysis. Bioinformatics.

[9] Zhao Z, Liu H (2008). Multi-source feature selection via geometry-dependent covariance analysis. JMLR Work Conf Proc.

[10] Tang J, Hu X, Gao H, Liu H. Unsupervised feature selection for multi-view data in social media. In: Proceedings of the 2013 SIAM International Conference on Data Mining.2013. p. 270–8.

[11] Feng Y, Xiao J, Zhuang Y, Liu X (2013). Adaptive unsupervised multi-view feature selection for visual concept recognition. Computer Vision–ACCV 2012.

[12] Wang H, Nie F, Huang H. Multi-view clustering and feature learning via structured sparsity. In Proceedings of the 30th International Conference on Machine Learning. 2013:352–60.

[13] Friedman J, Hastie T, Tibshirani R. A note on the group lasso and a sparse group lasso. arXiv preprint **arXiv:1001.0736**. 2010.

[14] Peng J (2010). Regularized multivariate regression for identifying master predictors with application to integrative genomics study of breast cancer. Ann Appl Stat.

[15] d’Aspremont A, Ghaoui LE, Jordan MI, Lanckriet GR (2007). A direct formulation for sparse pca using semidefinite programming. SIAM Rev.

[16] Lu M, Huang JZ, Qian X (2016). Sparse exponential family principal component analysis. Pattern Recog.

[17] Leek JT, Store JD (2007). Capturing heterogeneity in gene expression studies by surrogate variable analysis. PLoS Genet.

[18] Gagnon-Bartsch JA, Speed TP (2012). Using control genes to correct for unwanted variation in microarray data. Biostatistics.

[19] Lu M. An embedded method for gene identification in heterogenous data involving unwanted heterogeneity. In Proceedings of the 2018 IEEE International Conference on Bioinformatics and Biomedicine. 2018:242–7.

[20] Trevor H, Robert T, Andreas B (1994). Flexible discriminant analysis by optimal scoring. J Am Stat Assoc.

[21] David W, Srikantan N (2010). Iterative reweighted l1 and l2 methods for finding sparse solutions. IEEE J Sel Top Sign Process.

[22] Cope LM, Irizarry RA, Jaffee HA, Wu Z, Speed TP (2004). A benchmark for affymetrix genechip expression measures. Bioinformatics.

[23] Irizarry RA (2003). Summaries of affymetrix genechip probe level data. Nucleic Acids Res.

[24] Irizarry RA, Hobbs B, Collin F, Beazer-Barclay YD, Antonellis KJ, Scherf U, Speed TP (2003). Exploration, normalization, and summaries of high density oligonucleotide array probe level data. Biostatistics.

[25] Vawter MP (2004). Gender-specific gene expression in post-mortem human brain: Localization to sex chromosomes. Neuropsychopharmacology.

[26] Eisenberg E, Levanon EY (2003). Human housekeeping genes are compact. TRENDS Genet.

[27] Baird S, Fitch D, Kassem I, Emmons S (1991). Pattern formation in the nematode epidermis: determination of the arrangement of peripheral sense organs in the c.elegans male tail. Development.

[28] Tan J (2014). Integrative epigenome analysis identifies a polycomb-targeted differentiation program as a tumor-suppressor event epigenetically inactivated in colorectal cancer. Cell Death Dis.

[29] Agus DB, Bunn PA, Franklin W (2000). Her-2/neu as a therapeutic target in non-small cell lung cancer, prostate cancer, and ovarian cancer. Semin Oncol.

[30] Oh JJ, Grosshans DR, Wong SG (1999). Identification of differentially expressed genes associated with her-2/neu overexpression in human breast cancer cells. Nucleic Acids Res.

[31] Pal P, Xi H, Sun G, Kaushal R, Meeks J, Thaxton C (2007). Tagging snps in the kallikrein genes 3 and 2 on 19q13 and their associations with prostate cancer in men of european origin. Hum Genet.

[32] Nam R, Zhang W, Trachtenberg J, Diamandis E, Toi A, Emami M (2003). Single nucleotide polymorphism of the human kallikrein-2 gene highly correlates with serum human kallikrein-2 levels and in combination enhances prostate cancer detection. J Clin Oncol.

[33] Zhu C, Feng X, Ye G, Huang T (2017). Meta-analysis of possible role of cadherin gene methylation in evolution and prognosis of hepatocellular carcinoma with a prisma guideline. Med (Baltimore).

[34] Zhang B, Kirov S, Snoddy J (2005). Webgestalt: an integrated system for exploring gene sets in various biological contexts. Nucleic Acids Res.

